# Cyclists’ Anger As Determinant of Near Misses Involving Different Road Users

**DOI:** 10.3389/fpsyg.2017.02203

**Published:** 2017-12-15

**Authors:** Víctor Marín Puchades, Gabriele Prati, Gianni Rondinella, Marco De Angelis, Filippo Fassina, Federico Fraboni, Luca Pietrantoni

**Affiliations:** ^1^Human Factors, Risk and Safety, Department of Psychology, Università degli Studi di Bologna, Bologna, Italy; ^2^Transport Research Centre, Universidad Politécnica de Madrid, Madrid, Spain; ^3^Changing MObility (cambiaMO), Madrid, Spain

**Keywords:** anger, near misses, safety, cycling, bicycle, traffic

## Abstract

Road anger constitutes one of the determinant factors related to safety outcomes (e.g., accidents, near misses). Although cyclists are considered vulnerable road users due to their relatively high rate of fatalities in traffic, previous research has solely focused on car drivers, and no study has yet investigated the effect of anger on cyclists’ safety outcomes. The present research aims to investigate, for the first time, the effects of cycling anger toward different types of road users on near misses involving such road users and near misses in general. Using a daily diary web-based questionnaire, we collected data about daily trips, bicycle use, near misses experienced, cyclist’s anger and demographic information from 254 Spanish cyclists. Poisson regression was used to assess the association of cycling anger with near misses, which is a count variable. No relationship was found between general cycling anger and near misses occurrence. Anger toward specific road users had different effects on the probability of near misses with different road users. Anger toward the interaction with car drivers increased the probability of near misses involving cyclists and pedestrians. Anger toward interaction with pedestrians was associated with higher probability of near misses with pedestrians. Anger toward cyclists exerted no effect on the probability of near misses with any road user (i.e., car drivers, cyclists or pedestrians), whereas anger toward the interactions with the police had a diminishing effect on the occurrence of near misses’ involving all types of road users. The present study demonstrated that the effect of road anger on safety outcomes among cyclists is different from that of motorists. Moreover, the target of anger played an important role on safety both for the cyclist and the specific road users. Possible explanations for these differences are based on the difference in status and power with motorists, as well as on the potential displaced aggression produced by the fear of retaliation by motorized vehicle users.

## Introduction

In 2014, 2112 cyclists lost their lives in EU countries (European Road Safety Observatory, 2016). This corresponds to the 8.1% of the overall road deaths and represents an increase of the 0.3% as compared to the year before. In Spain, 2173 cyclists were involved in road accidents in 2015, of whom, 48 lost their lives (Dirección General de Tráfico, 2015). Moreover, there has been an increasing trend in the share of bicyclist fatalities regarding overall road fatalities in EU countries in the last few years (Evgenikos et al., 2016). To get a better understanding of the causes of road accidents, previous research has developed and employed surrogate measures (e.g., Davis et al., 2011; Wu and Jovanis, 2012; Laureshyn et al., 2017), among which near misses can be found. The theoretical background that allows the choice of surrogate measures for the study of crashes is based on the existence of an inverse relation between the severity of a safety outcome and its frequency (Laureshyn et al., 2017). Near misses are incidents, which differ from accidents regarding the severity of the outcome, with the former not involving personal or property damage. Moreover, near misses have been found to share causation with crashes, nevertheless, such two occurrences differ in the fact that, in near misses, the harmful outcome has been avoided (Güttinger, 1982; Davis et al., 2011). Indeed, previous research has found the number of crashes to be related to that of near misses and to be a smaller proportion of them (Powell et al., 2007; Marín Puchades et al., 2017). Given the higher frequency of near misses, they constitute a rich source of knowledge for accident research (Davis et al., 2011). Furthermore, there is evidence that near miss incidents are frequent and act as a considerable disincentive to cycling (Aldred and Crosweller, 2015).

Road users’ anger has been found to be a crucial variable for the understanding of safety related outcomes and behavioral adaptation leading to them (Lewis-Evans et al., 2013; Demir et al., 2016), nevertheless, the majority of the studies conducted so far have focused only on drivers’ anger (i.e., as opposed to other road users’), which has been defined as the tendency to become angry while driving (Deffenbacher et al., 1994; Demir et al., 2016). Previous research has found a positive relationship between car driver’s anger and aggressive road behavior, as well as between them and crash and safety related outcomes (e.g., Deffenbacher et al., 2002, 2003; Mesken et al., 2007; Bjorklund, 2008; Deffenbacher, 2008; Stephens and Ohtsuka, 2014; Stephens and Sullman, 2015). Zhang and Chang (2016) conducted a meta-analytic review which encompassed 51 studies that had been undertaken in the previous 20 years, and found that drivers’ anger was associated with both near misses and crashes, as well as with aggressive driving, driving errors and risky driving. Moreover, the authors stated that driver anger was most strongly related to aggressive driving, which has been found to mediate the relationship between anger and crash-related outcomes (Stephens and Sullman, 2015) and to be associated with feelings of frustration and hostile appraisals of other drivers (Gulian et al., 1989; Sümer, 2003). Demir et al. (2016) analyzed 48 studies using meta-analytic techniques from the scope of the Contextual Mediated Model (Sümer, 2003), which states that accident involvement (and thus, involvement in safety related outcomes) is predicted by the distal context, comprising road, vehicle and individual characteristics, and this relationship is mediated by the proximal context, consisting of driver’s performance and behavior. They found that driving anger was associated with outcome variables such as aggressive driving, errors and violations, consistent with Zhang and Chang (2016). Nevertheless, Demir et al. (2016) found that violations had the strongest relationship with driving anger.

Cyclists’s anger, as well as drivers,’ can come from different sources and have diverse motives. While investigating driving anger, receiving citations from police has been identified as a source of it (Deffenbacher et al., 2016). Since cyclists may share infrastructure with several different road users, previous research has also studied anger derived from interaction with some of them, among which car drivers, cyclists and pedestrians (Oehl et al., 2016). The aim of the present study is to investigate the effect of cyclists’ general anger, as well as anger directed to specific road users, on near misses involving them, as well as overall near misses.

According to the appraisal-tendency framework (Lerner and Keltner, 2000, 2001), emotion-specific appraisal processes trigger changes in cognition, physiology, and behavior. Specifically, fear and anger have opposite effects on risk perception and risk behaviors (Lerner and Keltner, 2001). Whereas fear tends to cause pessimistic risk estimates and risk-averse choices, anger leads individuals to perceive less risk and to risk-seeking choices. In addition, each emotion is associated with specific appraisals reflecting the central meaning of the event. Following a process defined as appraisal tendency, an emotion elicits a predisposition to appraise future events and predisposes individuals to act in specific ways that are coherent with the central appraisal dimensions associated with the emotion. A sense of individual control and certainty characterizes the appraisal-tendency of anger (Lerner and Keltner, 2001).

In the traffic context and following the appraisal-tendency framework (Lerner and Keltner, 2000, 2001), we can predict that angrier cyclists will be more likely to report near misses because of the effects of anger on risk perception and risk-seeking behavior. Indeed, appraisals of elevated certainty and individual control associated with anger should lead angry cyclists to perceive lower risk and adopt riskier behaviors. These behavioral changes in response to anger follow the principle of behavioral adaptation. Risk-adaptation theory combines three previous theories explaining how people adapt their behaviors to perceived risks (Koornstra, 2009). This theory states that individuals do not react to risks below an adaptive ideal risk threshold, which changes according to experienced risks and their consequences. Moreover, other authors have proposed that instead of having an ideal risk threshold against which road users compare the currently perceived risk, there is a target feeling that road users try to attain and keep (Vaa, 2013).

The inclusion of a target feeling as determinant of behavior is far from simple. The target feeling, and thus the actions to attain it, might vary as a result of the risks involved in the scenario as well as the anger provoked in the cyclist, since anger can increase perception of control and reduce that of risk. Moreover, anger leads to road aggression (Demir et al., 2016), and power imbalance can take a key role in this relationship. Cavacuiti et al. (2013) found that road users inside a vehicle felt more secure, with more power, and that power imbalance seemed to enhance how threatening potentially harmful actions or road aggression were perceived by the road user with less power.

Cyclists are considered vulnerable road users for several reasons (Prati et al., 2017). For instance, compared to motorists, cyclists face higher risks posed by motorized vehicles and are exposed to potentially more severe consequences due to differences in mass and speed, and to the lack of protection within a vehicle. Therefore, the previously stated effect of anger on both near misses and risky driving among car drivers (i.e., by lowering the ideal risk threshold) cannot be taken for granted for cyclists. Thus, depending on the relative power that cyclists hold in comparison with the road user with which one they interact, cyclists might be expected to feel a different level of risk (i.e., considering also the risk of being attacked or insulted) and to react differently to situation producing anger. When there is no such power imbalance, or it counts in favor of the cyclist, there might be higher chances that the cyclist responds with aggressive behavior to a situation that produces anger. Nevertheless, in cases of large power imbalance, where fear of punishment by the more powerful road user is present, anger provoked in the less powerful road users might not be translated into aggression toward the provoking agent, but into displaced aggression. Specifically, displaced aggression consists in producing aggression toward a target that is not the source of anger or frustration (Miller, 1941; Marcus-Newhall et al., 2000). Thus, cyclists that have experienced an enraging situation provoked by a car driver will try to attain the target feeling, but anger might not lower perceived risk enough to engage in aggressive behavior toward the car driver, this way cyclists might end up directing their aggression (i.e., derived from frustration) toward another road user that does not elicit fear of retaliation. Furthermore, the effects of power imbalance may not only apply to road users, but also to interaction with police officers, who have the power to punish the cyclists in case of violation of the road code. In this case, though, anger might not be expected to lead to any displaced aggression nor near misses since this would just generate more scenarios to interact with the police and probably get fined.

Based on the appraisal-tendency framework and risk adaptation theory, we hypothesize that higher cycling anger levels will be related to higher probability of being involved in near misses in general (Hypothesis 1) due to reduction of risk perceived associated with anger. We foresee that anger toward interaction with pedestrians and cyclists will increase the probability of being involved in near misses with pedestrians (Hypothesis 2) and cyclists (Hypothesis 3), respectively. The rationale behind consists in the power balance between cyclists and the imbalance favoring them when it comes to pedestrians that leads to think that anger will be associated with lowered risk perception and risk taking to achieve the target feeling, as well as with potential aggressive behavior toward them. On the contrary, we hypothesize that anger toward the interaction with cars will have a deterrent effect regarding near misses with cars (Hypothesis 4) due to power imbalance and the potentially severe consequences of involvement in such event and how these might also generate fear, which is associated with risk-aversion. We hypothesize that anger toward the interaction with the police will predict lower probability of being involved in near misses in general and with all sorts of road users (Hypothesis 5). The rationale for this resides in the power imbalance and the fact that unpleasant consequences of interaction with police (e.g., being fined) can take place regardless of the other road user since it depends on the cyclist’s behavior. Moreover, we intend to explore the effect of anger toward the interaction with different road users on the occurrence of near misses with other road user types.

To our knowledge, no previous research has addressed the consequences of cyclists’ anger. Part of the novelty of this study lies in that the context of cycling is divergent from that of car drivers insofar as cyclists are considered vulnerable and minority road users (Prati et al., 2017).

## Materials and Methods

### Procedure

An online survey was used to explore and understand the occurrence of near misses among cyclists in Spain and the implication of cycling anger on it. The research took place within the scope of the XCYCLE project^1^, a study investigating the safety of cyclists in their interaction with motorized vehicles. The study attempted to replicate an original design already validated in the literature (i.e., Aldred and Crosweller, 2015).

Data collection was carried out using the software Qualtrics Research Suite and it spanned from the 1st of March 2017 to the 8th of June 2017. We chose these 3 months period because cycling use appears to be higher during spring time (Thomas et al., 2012).

Study participants were a convenience sample of people who cycled – independently of the frequency and the purpose of their cycling – recruited all over Spain through various channels including cyclist organization and associations, announcement at cycling events and social media dissemination (e.g., twitter, Facebook). No incentives to participate in the survey were offered.

The questionnaire had two parts. On the first one, cyclists were invited to take part in the study by accessing a link provided by email, and registering by writing down their email address and agreeing to be contacted back on the day specified by them. Moreover, participants were informed that their email addresses would just be used to send them the link to the second part of questionnaire. This strategy was thought to minimize the drop out by nudging participants to respond the same day they used the bicycle. This is in line with the recruitment method proposed by Aldred and Crosweller (2015). On the second part of the questionnaire, each participant would receive an email – at the end of the day they had specified on the first part – with the link to the online survey where they could provide information about their cycling trips and any incidents that had taken place. Before beginning to reply to the questionnaire, participants were informed that their data would be treated anonymously and for the sole purpose of academic research. Moreover, participants were not invited to cycle to respond to the questionnaire, but to respond to the questionnaire on a date in which they were going to use the bicycle. Therefore, participating in the study did not involve any harm or risk that was not present had they not participated. Participants could complete the survey more than once (i.e., about more than one day of cycling), indicating if it was the first time they were replying to the questionnaire. Furthermore, if participants had not finally used the bicycle the day they had specified on the first part of the questionnaire, they were given the opportunity to specify another date so that they could receive the email with the link to the second part of the questionnaire again.

### Measures

The web-based questionnaire was developed with the clear intent to be used as a daily diary in which the cyclist could record their trips made during the day as well as experiences of near misses. The questionnaire contained questions about cyclists’ trips during their day, cyclists’ experience of possible near misses and cyclists’ anger toward different group of road users, as well as questions on cyclists’ demographics and experience.

#### Number of Trips

Participants were asked to record the number of trips they had completed by bicycle throughout the day from 0 to 10. This variable was later used as an indicator of individual exposure.

#### Near Miss

Participants were given the definition of near miss as any situation that happens while cycling where the person has felt fear or discomfort, excluding accidents resulting in physical or material damage. Then, participants were asked to provide the number of near misses that they had experienced that same day with the following item: “how many near misses have you had during your trips?”

#### Cycling Frequency

Respondents were asked how many times they were using the bicycle on average per week. Participants could choose between “Never,” “Less than one day a week,” “One day a week,” “Two days a week,” “Three days a week,” “Four days a week,” “Five days a week,” “More than five days a week.”

#### Cyclist’s Anger

We used the Cycling Anger Scale (Oehl et al., 2016) to measure cyclists’ anger. This scale is composed of fours subscales measuring anger toward: (1) Police Interaction; (2) Cyclist Interaction; (3) Car Interaction; and (4) Pedestrian Interaction. According to the authors, satisfactory convergent validity was demonstrated via correlations with Driving Anger Scale (Deffenbacher et al., 1994) and State Trait Anger Inventory (Schwenkmezger et al., 1992). In addition, each subscale presented acceptable reliability indices in Oehl et al. (2016). The reliability indices obtained in the present study were: Police Interaction (Cronbach’s Alpha: 0.80), Cyclist Interaction (Cronbach’s Alpha: 0.73), Car Interaction (Cronbach’s Alpha: 0.81), and Pedestrian Interaction (Cronbach’s Alpha: 0.93). To explore how cyclist’s anger directed toward different road users and the police affected the occurrence of near misses, participants were asked to indicate their amount of anger (either experienced previously or that would be experienced in the future) elicited by situations included in a list using a 5-point Likert scale ranging from 1 (*nothing*) to 5 (*a lot*). Examples of situations were: “You are fined for cycling without lights,” “You are fined for cycling on the wrong side of the street,” “A cyclist heads very quickly toward you obstructing your path,” “A car does not give you the right of way when it should,” “Pedestrians walk along the cycle path.”

#### Cyclists’ Demographics and Experience

We also collected data regarding gender, age, and ownership of motor vehicles (e.g., car, motorcycle).

### Participants

A total of 377 case entries (i.e., including those that had replied to the questionnaire more than once) were stored in our database during the 3-months long recruitment campaign. To clean the data, we excluded: (1) those participants that had not finished the questionnaire; (2) those that said that had had more than 9 near misses the day of the questionnaire administration since a wrong understanding of the definition of near miss seemed to underlie such exaggerated number of incidents; and finally, (3) those that stated to have slept no hours the night before. After discarding these cases, 254 entries were kept with valid trip records. Resulting sample features socio-demographic and mobility characteristics are shown in **Table 1**.

**Table 1:** Descriptive statistics of the survey sample.

Variable	Category	Study sample (*N* = 254)	
		*N*	*%*
Gender	Female	49	19.3%
	Male	205	80.7%
	Missing values	0	0.0%
Age group	<20	4	1.6%
(years)	20–29	48	18.9%
	30–44	132	52.0%
	45–54	45	17.7%
	55–64	22	8.7%
	>64	1	0.4%
	Missing values	2	0.8%
Mode availability	Car owner (or having access to)	161	63.4%
	Motorcycle owner (or having access to)	23	9.1%
Cycling frequency	Less than one day a week	1	0.4%
	One day a week	11	4.3%
	Two days a week	23	9.1%
	Three days a week	27	10.6%
	Four days a week	35	23.8%
	Five days a week	48	18.9%
	More than five days a week	109	42.9%
	Missing values	0	0.0%

As it can be seen in **Table 1**, the majority of the participants of the study used the bicycle five or more days a week. Most of them were male and also most were aged between 30 and 44 years old.

**Table 2** displays the frequencies and percentages of participants per number of near misses suffered. Moreover, these data are also shown for near misses involving each type of road user.

**Table 2:** Frequency and percentages of near misses involving each type of road users.

Number of near misses	Involving any road user	Involving cars	Involving bicycles	Involving pedestrian
0	113 (44.5%)	140 (55.1%)	233 (91.7%)	214 (84.3%)
1	68 (26.8%)	72 (28.3%)	12 (4.7%)	28 (11.0%)
2	51 (20.1%)	33 (13.0%)	6 (2.4%)	9 (3.5%)
3	8 (3.1%)	5 (2.0%)	1 (0.4%)	1 (0.4%)
4	7 (2.8%)	2 (0.8%)	–	–
5	3 (1.2%)	–	–	–
6	2 (0.8%)	–	—	–
Missing	2 (0.8%)	2 (0.8%)	2 (0.8%)	2 (0.8%)

*The first number in each cell corresponds to the frequency of participants and the one between brackets to the associated percentage. The frequencies and percentages do not add up between columns because near misses could involve more than one type of road user.*

### Statistical Analysis

Near misses are events or occurrences that can be represented as counts, that is, with non-negative integers. Counts are often modeled using the Poisson distribution, which assumes that the expected value and the variance are equal (Stroup, 2013). Equation 1, displayed below, represents the Poisson probability distribution function (Stroup, 2013; Faraway, 2016):

(1) $$\begin{array}{c} f(y)=\frac{e^{-\lambda }\lambda^{y}}{y!}\;\left(1\right) \end{array}$$

The previous equation models the probability that the number of events occurred by a specific interval of time (i.e., a ratio represented by *y*) take place given the mean ratio of events by that same interval. The Greek letter λ represents this mean value (and variance) of the distribution.

A Poisson regression model can be represented as in Equation 2, being X_1_, X_2_…X_n_ the independent variables and b_1_, b_2_…b_n_ the beta coefficients that indicate the change in the natural logarithm of the outcome (i.e., ln(y)) by each change in one unit of the independent variable (Coxe et al., 2009).

(2) $$In(y)\;=\;b_{0}\,+\;b_{1}X_{1}\,+\;b_{2}X_{2}\,\;+\;...\,+\;b_{n}X_{n}$$

Moreover, Equation 3 shows a representation of the Poisson regression model that is equivalent to that in Equation 2, nevertheless, this one allows to represent the changes in the dependent variable (i.e., y) and not on its transformation **[i.**e., ln(y)]. This way, in the results section, we have reported the beta values (i.e., *β*), that is, the effects on the logarithmic transformation of the dependent variable (i.e., Equation 2), as well as the Exp(*β*), which correspond to changes in the dependent variable (i.e., Equation 3).

(3) $$\begin{array}{c} y=e^{\left(b_{0}+b_{1}X_{1}+b_{2}X_{2}+...+b_{n}X_{n}\right)}\;\left(3\right) \end{array}$$

Poisson regression has been used to model the effect of several predictor variables on a count variable. This type of regression corresponds to a family of Generalized Linear Models, which allows for estimation of linear regression models with dependent variables that do not follow a normal distribution (Faraway, 2016).

Given that the main assumption for testing a Poisson model is that the distribution of the counts has a mean and variance that are equivalent, overdispersion can represent a threat to the estimation and interpretation of the models (Stroup, 2013). Nevertheless, this issue can be managed with slight modifications in the models (Stroup, 2013; Faraway, 2016).

A Kolmogorov–Smirnov test was used to explore whether the dependent variables followed a Poisson distribution.

To perform the analyses, we set the natural logarithm of the number of trips done by bicycle during that same day as the scale weight variable, that is, as a measure of exposure that influences the probability of being involved in near misses. Moreover, we controlled for the effect of age, gender and weekly bicycle use to rule out the variance explained by them that could be attributed to anger otherwise.

All the analyses were performed using SPSS version 23 and confidence interval levels of 95%.

## Results

The total number of near misses (*z* = 1.208, *p* > 0.5), and the near misses involving a car (*z* = 0.454, *p* > 0.05), a bicycle (*z* = 415, *p* > 0.05), and a pedestrian (*z* = 0.412, *p* > 0.05) followed a Poisson distribution.

In Hypothesis 1, we foresaw that cycling anger would be positively related to the probability of experiencing near misses. General cycling anger was not associated with the total number of near misses [*β* = -0.037, 95% Wald CI -0.302| 0.228; Exp(β) = 0.964, 95% Wald CI 0.740| 1.257], thus, not providing support for Hypothesis 1. The Omnibus Test showed that the model did not have better fit than an intercept only model, *χ^2^*(4) = 5.462, *p* = 0.243.

To explore how cyclist’s anger directed toward different road users and the police affected the occurrence of near misses, we performed a Poisson regression setting each one of the subscales of the Cycling Anger Scale as covariates and estimating their main effects on near misses. Moreover, to test the remaining hypotheses we performed the same analyses with near misses involving cars, bicyclists, or pedestrians as dependent variables. The estimates are displayed in **Table 3**. Anger toward the interaction with pedestrians increased the probability of having near misses with pedestrians (i.e., providing support for Hypothesis 2). Anger toward the interaction with cyclists did not affect the probability of being involved in near misses with cyclists (i.e., thus not providing support for Hypothesis 3), nor with any other road user type. Anger toward car interaction was found to increase the probability of experiencing near misses involving bicycles and pedestrians, whereas it did not affect the probability of experiencing near misses involving cars, thus not providing support for Hypothesis 4. Finally, anger toward interaction with the police decreased the probability of being involved in near misses with any of the road user types, but not in general, thus, providing partial support for Hypothesis 5.

**Table 3:** Estimates with Model Fit for the Multiple Poisson Regressions.

	Near miss	Near miss with cars	Near miss with bicycle	Near miss with pedestrians
Anger car interaction	1.181 (0.877–1.590)	1.229 (0.889–1.699)	**3.357 (1.527–7.380)**	**1.812 (1.133–2.899)**
Anger bicycle interaction	0.979 (0.786–1.219)	0.888 (0.696–1.133)	0.967 (0.648–1.443)	0.855 (0.632–1.157)
Anger pedestrian interaction	1.056 (0.899–1.240)	1.100 (0.921–1.313)	1.059 (0.780–1.438)	**1.535 (1.206–1.952)**
Anger police interaction	0.871 (0.748–1.014)	**0.825 (0.697–0.977)**	**0.753 (0.572–0.992)**	**0.768 (0.619–0.954)**
Omnibus Test (χ^2^(*p* value))	9.401 (*p* = 0.225)	13.774 (*p* = 0.055)	26.190 (*p* < 0.001)	34.945 (*p* < 0.001)

*The values in the rows of the anger subscales correspond to the Exp(β) (CI). Values bigger than 1, with CI not including it, represent an increase in probability due to increases of the independent variable and vice versa. Values in bold are statistically different than 1. The four subscales where included as predictors in the model contemporarily.*

## Discussion

The aim of the present study was to explore if anger experienced by cyclists in different situations involving road users and police had an effect on the occurrence of near misses in general and with the different road users. The major novelty of the current study is that we investigated the influence of anger on a safety related outcome for the first time in a cyclists’ population. Furthermore, anger is considered as hetero-directed, meaning we deepened the understanding of anger as a social element, or in other words, as a feeling that arises in the social context (i.e., the road system in our case) when interacting with other road users. Specifically, we examined the effect of anger toward different categories of road users and police on near misses with such road user types, as well as with other road users.

Results regarding Hypothesis 1 showed that cycling anger did not predict any changes on the probability of near misses. This differs from previous research studying car drivers’ anger which found a positive association between driving anger and involvement in crash related outcomes (Demir et al., 2016; Zhang and Chang, 2016). In fact, in previous studies, drivers admitting to having more anger while driving also tended to have higher accident records. For instance, Delhomme et al. (2012) gathered a sample of 2038 young drivers and found a significant association (i.e., correlation) between anger and the number of crashes experienced. Moreover, Deffenbacher et al. (2003) performed a comparison between groups of high and low anger drivers (*N* = 153) and found a significant effect of the level of anger on crash related outcomes, such as near misses and moving violations. These differences in findings might be due to the fact that the scale used to measure anger comprised anger toward a defined set of road users and interactions with police, whereas near misses included manifold situations that might just not be related to the interactions encompassed in the scale (e.g., single near misses due to slippery road).

Regarding the rest of the hypotheses, the present study demonstrated that anger toward the interaction with pedestrian leads to a slight increase in the occurrence of near misses with pedestrians (Hypothesis 2) but not with bicycles nor cars. This is in line with previous evidence in the driving field according to which anger leads to more aggression and riskier behaviors (Demir et al., 2016). Feeling anger (in this case toward pedestrians) may lead to experiencing a detriment in one’s range of cognitive performance such as attention allocation, reasoning, judgment, and decision making, thus perceiving a lower level of risk while at the same time showing a more favorable attitude toward risk taking (Blanchette and Richards, 2010).

Anger toward other cyclists was not associated with near misses involving cyclists (Hypothesis 3) nor with other types of road users. We have found two possible explanations for these findings. First, although cyclists might be considered to have the same status with regard to other road users, there might be individual differences in the perception of their status regarding their peer cyclists, that is, some cyclists might perceive they have higher or lower status than other particular cyclists. Thus, anger toward cyclists might lead to contradictory effects on aggression and risk taking depending on cyclists and their perceived status in general and regarding specific cyclists with whom they might interact. The second possible explanation regards the fact that cyclists might share a social identity and feel part of an in-group (Prati et al., 2017), thus, even if different levels of anger might be felt among them, this does not lead to any sort of aggression or riskier behavior involving other cyclists.

Furthermore, the results showed that cyclists who reported anger toward the interaction with cars do not have an increased probability of experiencing a near miss with cars (Hypothesis 4), but they do with cyclists and pedestrians. This could be due to displaced aggression (Marcus-Newhall et al., 2000). Thus, the more the anger felt toward interactions with cars, the more cyclists might avoid them and, due to power imbalance, displace their aggression (i.e., as outcome of anger) against other cyclists and pedestrians with whom they might share cycling lane or sidewalk.

Above all, results of the present work showed a strong negative effect of anger toward the interaction with police forces on the probability of experiencing near misses with each one of the road users (Hypothesis 5). Cyclists who are angry, afraid, and frustrated when interacting with police could probably do whatever is in their power to avoid the interaction with law enforcers. This most likely means that cyclists will avoid committing violations, risky-riding, and aggressive riding on public roads to avoid being fined, thus riding more carefully and consequently reducing their probability of experiencing near misses involving other road users. Nevertheless, such anger might not affect near misses that involve only the cyclist that experiences it, thus wielding no influence on general near misses, which include all sorts of situations. Moreover, there is an essential difference between interaction with other road users and police as to how it has been measured: interaction with police does not refer to events that take place genuinely, but to being fined after having committed a violation.

These results provide support for the idea that anger is proximal with respect to crash related outcomes. Past psychological models focused mostly on personality traits, emotional and behavioral components of road anger (Britt and Garrity, 2006; Stephens and Sullman, 2015). We argue that a social component and the consideration of power differences between road users should be included in future models to understand the complexity of anger in the road context and how it is related to negative outcomes. We propose that feeling angry due to another road user with equal or lower power might foster risk taking, and this can lead the person to commit aggressive cycling, risky cycling and errors, all aberrant behaviors which have been found to correlate with anger (Zhang and Chang, 2016). Those aberrant behaviors, for example tailgating and cutting the road in driving, will most likely provoke irritation in other road users, triggering a vicious circle that is related to increased anger toward the other road users and a desire for retaliation (Bjorklund, 2008). As Bjorklund (2008) concluded, experienced irritation often leads to openly aggressive actions, and that expression of aggressive behaviors may be a cause of other drivers’ feeling of irritation. To support this argument, we can cite the Cognitive-Neoassociation Theory of aggression (Berkowitz, 2012) as well as the General Affective Aggression Model (Anderson, 1997), which state that aggression comes from anger-related affective states. In turn, anger-related affective states can be instigated by unfair behaviors that lead to being disappointed by the imbalance between expectations and the behavior (Shinar, 1998). Furthermore, when cyclists feel angry about an interaction with a road user with more power, such power imbalance may lead to avoidance of those interactions and displaced aggression toward other road users with equal or lower power. This suggests that cyclists could benefit from road segregation since that would reduce displaced aggression toward themselves. The present study fosters the idea that, to deepen the understanding of the phenomenon, future studies on road anger should include a social perspective.

One of the limitations of this study regards the fact that the sample gathered was a convenience sample, that is, it has been drawn from a part of the population that was more accessible, in this case, due to their activity in social media or cyclists’ associations. Thus, results are not generalizable to all the population. Another limitation of this study concerns the Common Method Variance, which consists in the variance between variables that is explained by the use of the same method (Podsakoff et al., 2003). In other words, some of the variance found between independent and dependent variables might be attributable to using the same method to measure them (i.e., survey).

Future research should focus on clarifying the relationship between anger toward car drivers and the increase in probability of near misses with cyclists and pedestrians. A conceivable way to address this question could be a combination of questionnaire measuring anger with GPS coordinates of trips to know if anger toward the interaction with car drivers leads to avoidance by further using of sidewalks or cycling lanes. Moreover, naturalistic cycling studies could give an answer to this question as well as provide more details as to how these near misses take place, providing a richer explanation of the steps that lead anger toward car drivers to more near misses with cyclists. In addition, due to the differences in power status and mass between cyclists and other road users, future studies should address how the relationship between trait anger and near misses can be explained by increments in aggressive cycling, as suggested by the driving literature (e.g., Demir et al., 2016), in combination with avoidance of a threat in the case of vehicles with greater mass (e.g., cars, trucks) and displaced aggression toward road users with equal or lower power. Moreover, given the results regarding interaction with the police, it would be interesting to study differences in near misses and unsafe cycling behaviors between countries with different levels of intensity of enforcement about cycling violations.

## Conclusion

Given the objectives of this study, Poisson regression results showed how cycling anger affected near misses’ occurrence on the basis of the source of anger and the road users involved in the near misses. Anger toward pedestrian has been shown to have a slight positive effect on the probability of near misses with pedestrians. In contrast with our hypothesis, cycling anger toward car drivers did not have a deterrent effect on the probability of near misses with them, but has a positive effect on near misses with other cyclists and pedestrians. Moreover, anger toward the interaction with police was associated with fewer near misses in the interaction with all types of road users.

Results of our study provide evidence for cyclists’ anger effects on road safety outcomes. Our findings support the differentiation between the effects of drivers and cyclists’ anger, deepening the knowledge and understanding on anger in the traffic context. In this regard, we argue that a social component of anger should be considered and introduced in future models and researches. Finally, the outcomes of the present studies are relevant from a theoretical point of view, laying the foundations for future studies on cyclists’ anger effects on traffic safety. The novelty of the present study is that it is the first to explore the effects of cyclists’ anger.

## Ethics Statement

The present study adhered to the principles of informed consent, benefit and not harm, and confidentiality. Participants were notified about the treatment of their data for research purposes only and were made aware of providing their consent by continuing to reply to the online questionnaire. Moreover, participating in the study did not involve any action nor visualization of content of any type, and they only had to respond questions about their experiences that day. Since they were not asked to use the bicycle to respond to the questionnaire, but to respond to the questionnaire when they had used the bicycle, we could exclude that any potential harm that they could suffer while cycling was due to the participation in the study (i.e., since they used the bicycle regardless of the study).

## Author Contributions

All authors listed have made a substantial, direct and intellectual contribution to the work, and approved it for publication.

## Conflict of Interest Statement

The authors declare that the research was conducted in the absence of any commercial or financial relationships that could be construed as a potential conflict of interest.

## Footnotes

1 http://www.xcycle-h2020.eu/
